# Context-dependent role of trisomy 6 in myelodysplastic neoplasms and acute myeloid leukemia: a multi-omics analysis

**DOI:** 10.1038/s41375-024-02268-w

**Published:** 2024-05-11

**Authors:** Hussein Awada, Arda Durmaz, Tariq Kewan, Fauzia Ullah, Danai Dima, Hassan Awada, Simona Pagliuca, Manja Meggendorfer, Torsten Haferlach, Carmelo Gurnari, Valeria Visconte, Jaroslaw P. Maciejewski

**Affiliations:** 1 Department of Translational Hematology and Oncology Research, Cleveland Clinic, Cleveland, OH USA; 2 Yale Cancer Center, New Haven, CT USA; 3 Roswell Park Comprehensive Cancer Center, Buffalo, NY USA; 4 Department of Clinical Hematology, CHRU de Nancy, Nancy, France; 5 MLL Munich Leukemia Laboratory, Munich, Germany; 6 Department of Biomedicine and Prevention, University of Rome Tor Vergata, Rome, Italy

**Keywords:** Acute myeloid leukaemia, Myelodysplastic syndrome

## To the Editor:

Karyotypic aberrations of chromosome (chr) 6 include duplications (trisomy 6 [+6]), deletions of its short arm (del6p), uniparental disomy 6p (UPD6p), as well as various microduplications and microdeletions. Lesions involving 6p are of particular interest as they point toward HLA locus involvement, including loss of heterozygosity and/or haploinsufficiency, as pathogenic drivers resulting from deletions and UPD6p [1, 2]. Along with somatic mutations in various HLA alleles, deletions of various sizes including microdeletions of the HLA locus as well as somatic UPD6p have been suggested to result from immune pressure and to act as means of escape hematopoiesis in immune-mediated aplastic anemia (AA) [3, 4]. Similarly, our group described these lesions in the context of loss of graft versus leukemia (GvL) effect of mismatched donor HLA alleles following allogeneic hematopoietic stem cell transplant (alloHSCT) with subsequent relapse of myeloid leukemias [5]. We further demonstrated that the enrichment of specific amino acids within the peptide-binding groove of HLA class II, especially HLA-DRB1, affects its interaction with the T-cell receptor (TCR) and hence underlies the autoreactivity inherent to autoimmune AA [6].

Trisomy 6 has been observed in myelodysplastic neoplasm (MDS) and acute myeloid leukemia (AML) and can occur as a sole genomic abnormality. It has also been reported in AA and other bone marrow failure disorders [7]. However, to date reports have been limited to individual or small case series, hence precluding a comprehensive analysis of the clinical and molecular features of these patients. Specifically, the pathogenesis of duplication has not been clarified and may involve various potential oncogenes located on chr 6, e.g.*, RAB44*, *ECT2L*, among others. Alternatively, as with other chromosomal lesions, primary drivers may be located on other chromosomes. In addition, the intrachromosomal disruption of the HLA locus through microdeletions or loss of function mutations may possibly facilitate disease evolution and progression. While analyzing the HLA locus in AA, we observed several cases with isolated +6 (iso +6), which served as the impetus for the formulation of this report in lieu of the association of analogous occurrences in MDS and AML and the enigmatic clinical features and diverse pathogenesis of +6.

**Fig. 1:**
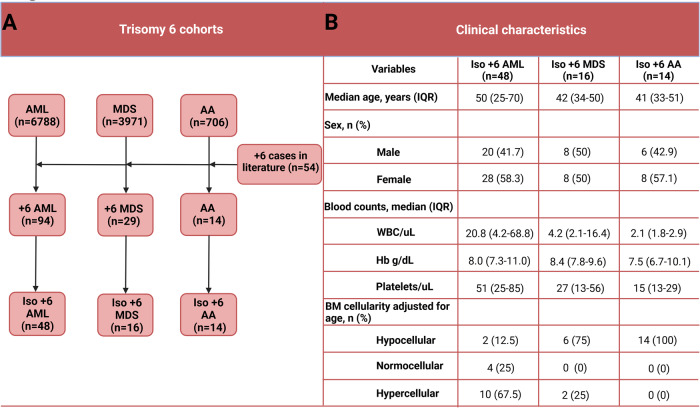
Summary and clinical characteristics of the included cases. **A** Flow diagram of patients with trisomy 6 and aplastic anemia (AA), myelodysplastic neoplasm (MDS), or acute myeloid leukemia (AML) included in our study. **B** Comparison of baseline clinical characteristics of isolated trisomy 6 (iso +6)-related AA, MDS, and AML using unpaired *t*-test for continuous variables and Chi-square or Fisher’s exact test for categorical variables.

In addition to cases from our clinics (*N* = 83), our search was complemented by iso +6 cases described in the literature (*N* = 54) to incorporate additional clinical data on iso +6 AA, MDS and AML [7] (metanalytic cases are summarized in Supplementary Tables S1 and S2). In total, we were able to examine a large cohort of patients collected from multiple sources and diagnosed with MDS (*n* = 3971), AML (*n* = 6788), or AA (*n* = 706) for iso +6, non-isolated +6 (non-iso +6), and normal karyotype (NK) disease for comparisons (Fig. 1A and Supplementary Appendix) [8–12]. For selected cases with +6 and healthy controls (*N* = 8), we have also performed expression analysis using bulk deep RNA-sequencing (RNA-seq) (Supplementary Appendix).

**Fig. 2:**
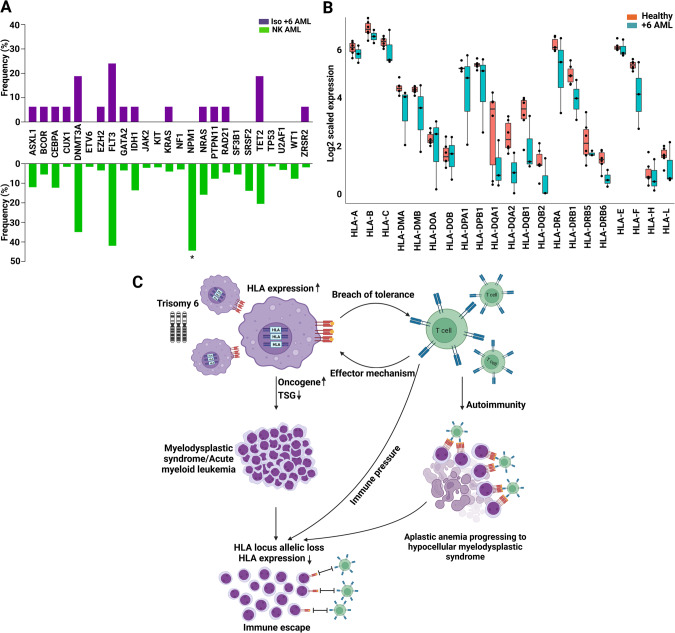
Genomic characteristics and proposed mechanisms of trisomy 6-mediated myeloid neoplasms. **A** Spectrum of mutations in trisomy 6 acute myeloid leukemia. The similar mutational patterns across iso +6 AML and NK AML except for *NPM1* mutations which are less common in isolated +6 AML. **B** The expression of HLA genes in trisomy 6 AML patients vs healthy controls. **C** Scheme illustrating the proposed dichotomy “autoimmunity vs immune pressure with HLA somatic loss” underlying the mechanisms of trisomy 6-related myeloid disorders.

In total, we identified 94 +6 AML patients, of whom 48 had iso +6 and 46 non-iso +6. In addition, 16 iso +6 MDS and 14 iso +6 AA cases were found (Fig. 1A and Supplementary Tables S1, S2). Of note is that, the iso +6 AML cases from our cohort (*n* = 16) and the one reported in the literature (*n* = 32) exhibited a similar clinical phenotype and survival characteristics (Supplementary Table S3). In general, iso +6 AML presented at a younger age of 50 vs 66.3 years (*P* < 0.0001) compared to NK AML. Iso +6 had analogous ontogenesis as NK AML (primary AML – pAML – proportions of 89.4 vs 85.7%, *P* = 0.7), and exhibited a similar hyperproliferative AML phenotype (median WBC count 20.8 vs 14.4 × 10^9^/L, *P* = 0.8). Targeted next-generation sequencing demonstrated similar co-mutational patterns to NK AML, including *FLT3* mutations, except *NPM1* mutations which were not found in iso +6 (0 vs 44.5% in NK AML, *P* = 0.0013; Fig. 2A). Remarkably, 5/16 iso +6 AML patients did not harbor any of the common myeloid mutations (Supplementary Table S4). The remaining cases of iso +6 AML were enriched for *DNMT3A*, *FLT3*, and *TET2* but had less *TP53* mutations compared to non-iso +6 disease (Supplementary Table S5). Despite the low mutational burden, iso +6 in AML patients had shorter overall survival (median 22 vs 32.1 months; *P* = 0.04) when compared to NK AML (Supplementary Fig. S1); yet, survival was not significantly different relative to non-iso +6 AML (22 vs 11 months, *P* = 0.1; Supplementary Fig. S1). In terms of treatment, 2/2 patients who received 7 + 3 regimens followed by cytarabine consolidation achieved remission, and 4/6 iso +6 AML patients successfully underwent alloHSCT and remained relapse-free post-transplant (Supplementary Table S3).

In MDS, iso +6 disease also presented at a significantly younger age vs NK MDS (median 43 vs 70 years, *P* < 0.0001). Interestingly, iso +6 MDS manifested a distinct clinical phenotype, notably characterized by profound anemia (median 8.4 vs 9.5 g/dL, *P* = 0.04) and hypocellular BM morphology (75% vs 9.5%, *P* = 0.0002) compared to NK MDS. Like AML, 3 of 4 iso +6 MDS patients with available sequencing did not have any detectable mutations. Transformation rate to AML was 37.5% with a median time to progression of 5 months (IQR 2–33.5). The association of +6 with pancytopenia and hypocellularity in 75% of the MDS cases is suggestive of a pathogenetic similarity with AA. Indeed, 2 of 14 patients with iso +6 AA eventually transformed to MDS.

When performed, RNA-seq in +6 AML (*n* = 3) vs healthy BM samples (*n* = 5) showed lower HLA mRNA expression in +6 AML, including 2/3 +6 AML cases with HLA expression ≤20th percentile of controls in 18 of 21 sequenced HLA genes, and even completely absent in 14 of the 21 HLA genes (Fig. 2B). We then analyzed HLA genes to search for somatic hits in +6 MDS/AML (*N* = 8) using our in-house developed HLA pipeline [6]. While no mutations were detected in the +6 MDS (*n* = 3) or +6 AML (*n* = 5) samples, yet 2/5 +6 AML patients were found to have allelic loss of HLA-DRB1 (Supplementary Table S6). In contrast, none of the 3 +6 MDS cases had any HLA lesions (Supplementary Table S6). Both +6 AML patients with allelic loss were pAML and had complex karyotypes, comparable age (67 vs 58 years), similar Hb (9.3 vs 9.4 g/dL) and platelet count (16 vs 14 k/uL), and a large percentage of BM blasts (79 vs 60%). However, the first patient had significantly higher WBC count (162.6 vs 5.3 k/uL) and higher number of co-mutations (*ASXL1, PRPF8, SRSF2* and *TP53* vs *BCOR* and *TP53*, respectively). With regard to outcomes, both patients survived <1 month after diagnosis.

Our results suggest that +6 may present as the sole cytogenomic abnormality in AA with progression to MDS as suggested by the hypoproliferative phenotype reminiscent of prior AA. It is possible that +6 via duplication of HLA locus may lead to increased expression of peptides triggering immune response in AA, e.g., as part of tumor surveillance reaction. In contrast, +6 in the context of AML exhibits adverse risk possibly due to paucity of prognostically favorable *NPM1* mutations. In addition, the absence of heightened HLA expression in certain +6 AML cases may contribute to the distinct clinical behavior of +6 in AML through HLA downregulation or segmental microdeletions as demonstrated in our study. Based on these results, one could speculate that an increased copy of the HLA locus in AA and MDS may lead to increased autoantigen presentation beyond TCR activation threshold, possibly leading to breach of tolerance, a mechanism that may be operative in AA and hypocellular MDS (Fig. 2C). Thus, clonal duplication through copy number gains, +6, or UPD of the HLA locus in MDS may result in immune pressure as seen in AA and GvL effect [13–15]. Indeed, we identified frequent combination features of pancytopenia and BM hypocellularity in iso +6 MDS in this series. In more advanced diseases, additional triggers such as deletions of tumor suppressor genes or acquisition of subclonal mutations may also play a role, with progression reflecting somatic pressure to overcome immune inhibition. Conversely, the allelic loss and lower expression of HLA in +6 AML may imply a dichotomy of mechanisms by which +6 contributes to advanced leukemia vs bone marrow failure. Further evidence of allelic deletion of HLA genes in +6 AML points toward immune escape and evasion, a mechanism already described in relapsing AML under immune pressure [5] and potentially contributing to the transformation of AA to malignant disease in this series.

To the best of our knowledge, we herein performed the most comprehensive clinico-genomic meta-analysis of the largest cohort ever reported of +6 in myeloid neoplasms, given its rarity in the literature. Our study includes novel analyses of the contrasting, context-dependent role of HLA in mediating the pathogenesis of MDS and AML by predisposing to hyperimmunity and its somatic alIelic loss, respectively.

### Supplementary information

Supplemental Material

## Data Availability

The authors have included relevant clinical and genetic data in the main text of the article and Supplementary Appendix. For additional information, please contact the corresponding author (maciejj@ccf.org).
