# Supplementary material for: Context-dependent role of trisomy 6 in myelodysplastic neoplasms and acute myeloid leukemia: a multi-omics analysis
Source: Leukemia. 2024 May 11;38(6):1411–4. doi: 10.1038/s41375-024-02268-w (PMC11147751; doi:10.1038/s41375-024-02268-w)
Supplement: Supplementary file 1 — Supplemental Material [file 41375_2024_2268_MOESM1_ESM.docx]

**Supplementary Appendix**

**Context-dependent role of trisomy 6 in myelodysplastic neoplasms and acute myeloid leukemia: a multi-omics analysis**

**Additional tables: 6**

**Additional figures: 1**

**References: 13**

**METHODS
Patients population.** Patients were assembled from multiple centers in addition to cases reported in the literature. Briefly, acute myeloid leukemia (AML) cases (n=6788) were combined from databases of the Cleveland Clinic Foundation (CCF) (n=855), The Munich Leukemia Laboratory (MLL) (n=4002), and publicly available datasets (including The BEAT AML master trial and The Cancer Genome Atlas (TCGA) program) (n=1931) to form a cohort of 6788 AML patients^1-3^**.** Myelodysplastic neoplasm (MDS) cases were collected from CCF (n=1627), MLL (n=1275), and The EuroMDS cohort (n=686) to form a cohort of 3588 MDS patients^1, 4^. Aplastic anemia (AA) patients were collected from CCF (n=706)^5^. Clinical and demographic data were collected from the electronic medical records of patients enrolled at participating institutions in accordance with the protocols and written consent approved by Institutional Review Boards and the Declaration of Helsinki. In addition, the Mitelman Database of Chromosome Aberrations and Gene Fusions in Cancer was interrogated for MDS and AML cases with trisomy 6 (+6)^6^. A total of 95 +6 AML patients were identified, of whom 49 were iso +6 and 46 non-iso +6. In addition, 16 iso +6 MDS and 14 iso +6 aplastic anemia patients were found and included in this study.

**Conventional cytogenetics*.*** G-banded cytogenetic analysis was performed on stored bone marrow (BM) samples of CCF and MLL patients when available using standard techniques. Cytogenetics of the publicly shared datasets were analyzed as reported by the original studies. Karyotypes were described according to the International System for Human Cytogenetic Nomenclature^7^.

**Genomic studies*.*** Molecular studies on CCF and MLL patients were collected through targeted sequencing panels including diagnostic next-generation sequencing (NGS) and RNA-sequencing techniques as previously described^8, 9^. Variants were annotated using Annovar and their and their somatic status was called using an in-house bio-analytic pipeline^10, 11^. All sequencing studies were performed on samples taken at initial diagnosis. HLA expression was determined by bulk deep RNA-sequencing libraries according to an Illumina paired-end library protocol. Briefly, isolated RNA samples that met appropriate cut-offs for both quantity and quality were quantified by qRT-PCR using a commercially available kit (KAPA Biosystems) and insert size distribution determined with the LabChip GX or Agilent Bioanalyzer. Sample concentrations were normalized to 1.2 nM and loaded onto an Illumina NovaSeq flow cell at a concentration that yields 50 million passing filter clusters per sample. Samples were sequenced using 100bp paired-end sequencing on an Illumina NovaSeq according to Illumina protocols. Data generated during sequencing runs were simultaneously transferred to the YCGA high-performance computing cluster. Primary analysis - sample de-multiplexing and alignment to the human genome - was performed using Illumina's CASAVA 1.8.2 software suite. Expression quantification was done using Salmon quantification with 100 bootstrap estimates correcting for position and gc bias^12^. Gene level summarization was done using R tximport package with median of the bootstrap estimates^13^. For HLA sequencing, AlloSeq Tx17 (CareDx) was used and mutational and copy number variation analysis performed as described elsewhere^5^.

**Statistical analysis.** Quantitative variables were expressed as median and interquartile range. Qualitative variables were expressed as numbers with their respective percentages. For all relevant group comparisons, unpaired two-sided t-tests were performed for comparisons of quantitative variables. Fisher’s exact test or Chi-square were used for testing of significance for qualitative variables. Kaplan–Meier (KM) log rank testing was used to calculate and test for significance of overall survival (OS) which was defined as the time from AML diagnosis to last follow-up or death for any cause.

All statistical tests were two-sided, and a P-value ≤ 0.05 was considered statistically significant. A P-value ≤0.05 was considered statistically significant for all types of analyses. All statistical computations and data visualizations were performed using R 3.6.2 ([www.r](http://www.r)-project.org) and Prism (GraphPad 9.0).

**Table S1. All cases of trisomy 6 from our centers included in the clinical analysis of the study.**

| **UPN** | **Diagnosis** | **Age (years)** | **Sex** | **Isolated +6** | **WBC (x10^9^/L)** | **Hb (g/dL)** | **Platelets (x10^3^/uL)** | **Bone marrow cellularity adjusted for age** |
| --- | --- | --- | --- | --- | --- | --- | --- | --- |
| **1** | pAML | 52 | M | Y | 60 | 6.8 | 36 |  |
| **2** | pAML | 83 | F | Y | 27 |  |  |  |
| **3** | pAML | 80 | F | Y |  |  |  |  |
| **4** | pAML | 48 | M | Y | 3.3 | 6.1 | 92 |  |
| **5** | pAML | 56 | F | Y | 20.1 | 11.4 | 113 |  |
| **6** | pAML | 68 | F | Y | 1.2 | 6.4 | 42 |  |
| **7** | pAML | 71 | M | Y |  |  |  |  |
| **8** | pAML | 83 | F | Y | 1.8 | 8 | 170 |  |
| **9** | pAML | 51 | M | Y | 2.2 | 8.4 | 25 |  |
| **10** | pAML | 54 | F | Y |  |  |  |  |
| **11** | pAML | 21 | F | Y | 62.6 | 7.5 | 32 |  |
| **12** | pAML | 70 | F | Y | 91.4 | 8.1 | 71 |  |
| **13** | pAML | 74 | F | Y | 75 | 9 | 80 |  |
| **14** | pAML | 29 | F | Y | 45 | 7.8 | 39 | Hyper |
| **15** | pAML | 70 | F | Y | 2.4 | 7.7 | 49 | Hyper |
| **16** | pAML | 66 | M | Y | 14.9 | 7.7 | 92 |  |
| **17** | pAML | 63 | F | N |  |  |  | Hyper |
| **18** | pAML | 40 | F | N | 4.9 | 9.2 | 21 | Hyper |
| **19** | pAML | 72 | M | N | 1.5 | 11 | 77 | Normo |
| **20** | pAML | 84 | F | N | 3.3 | 6.1 | 9 | Hyper |
| **21** | pAML | 64 | F | N | 18.2 | 8.7 | 34 |  |
| **22** | pAML | 74 | M | N | 0.9 | 8.9 | 100 | Hyper |
| **23** | pAML | 66 | M | N |  |  |  | Hyper |
| **24** | pAML | 80 | F | N | 1.5 | 9.1 | 137 | Hyper |
| **25** | pAML | 81 | M | N | 0.7 | 9.4 | 25 |  |
| **26** | pAML | 52 | M | N | 4.4 | 6.1 | 18 | Hyper |
| **27** | pAML | 69 | F | N | 50.3 |  |  |  |
| **28** | pAML | 73 | F | N | 81.2 | 7.1 | 18 | Hyper |
| **29** | pAML | 22 | F | N | 24.5 | 8.1 | 65 |  |
| **30** | pAML | 75 | M | N | 1.7 | 10.7 | 137 | Hyper |
| **31** | pAML | 65 | M | N | 112.2 | 6.8 | 53 | Hyper |
| **32** | pAML | 78 | F | N |  |  |  | Hyper |
| **33** | pAML | 44 | M | N |  |  |  | Hyper |
| **34** | pAML | 72 | F | N | 1.3 | 8.6 | 56 | Hyper |
| **35** | pAML | 72 | F | N | 189 | 6.7 | 73 |  |
| **36** | pAML | 75 | F | N | 1.1 | 8.5 | 65 | Hyper |
| **37** | pAML | 44 | F | N | 85 | 12.2 | 34 | Hyper |
| **38** | pAML | 90 | F | N | 12.3 | 13.2 | 78 |  |
| **39** | pAML | 89 | F | N | 4 | 8.4 | 3 | Hyper |
| **40** | pAML | 83 | M | N | 13.1 |  |  | Hyper |
| **41** | pAML | 60 | F | N |  |  |  |  |
| **42** | pAML | 27 | F | N |  |  |  | Hyper |
| **43** | pAML | 82 | M | N |  |  |  | Hyper |
| **44** | pAML | 80 | M | N | 2.9 | 8.1 | 21 |  |
| **45** | pAML | 72 | F | N | 0.6 | 5.8 | 80 | Hyper |
| **46** | pAML | 58 | M | N | 3.8 | 9 | 53 | Hyper |
| **47** | pAML | 58 | M | N | 58.1 | 10.8 | 155 | Hyper |
| **48** | pAML | 62 | M | N | 1.9 | 8.3 | 28 | Hyper |
| **49** | pAML | 72 | M | N |  |  |  | Hyper |
| **50** | pAML | 60 | F | N | 5.1 | 6.1 | 22 | Hyper |
| **51** | pAML | 67 | F | N | 0.9 | 9.6 | 149 |  |
| **52** | pAML | 58 | M | N | 5.3 | 9.4 | 14 | Hyper |
| **53** | pAML | 67 | F | N | 162.6 | 9.3 | 16 | Hyper |
| **54** | pAML | 83 | M | N | 2.3 | 9.5 | 97 | Normo |
| **55** | pAML | 22 | M | N | 48.5 | 8.2 | 15 | Hyper |
| **56** | pAML | 51 | M | N | 24 | 12.5 | 106 | Hyper |
| **57** | sAML | 66 | M | N | 4.5 | 9.3 | 21 | Hyper |
| **58** | sAML | 67 | F | N | 5.3 | 10 | 66 | Hypo |
| **59** | sAML | 72 | F | N | 3.5 | 7.2 | 14 | Hyper |
| **60** | sAML | 85 | F | N |  |  | 30 | Normo |
| **61** | sAML | 63 | M | N | 4.2 |  | 43 |  |
| **62** | sAML | 70 | M | N |  |  |  |  |
| **63** | MDS | 66 | F | N | 5 | 6.6 | 672 | Hyper |
| **64** | MDS | 19 | F | Y | 9.9 | 9.3 | 626 | Hyper |
| **65** | MDS | 85 | M | N | 2.7 | 9.8 | 62 | Hypo |
| **66** | MDS | 49 | M | N | 2.1 | 8 | 192 | Hyper |
| **67** | MDS | 71 | M | Y | 3.1 | 8.4 | 27 | Hypo |
| **68** | MDS | 62 |  | N |  | 10 | 145 |  |
| **69** | MDS | 77 |  | N |  | 12 | 121 |  |
| **70** | MDS | 72 |  | N |  | 8 | 30 |  |
| **71** | MDS | 43 |  | N |  | 8.8 | 88 |  |
| **72** | MDS | 82 |  | N |  | 9.8 | 132 |  |
| **73** | MDS | 69 |  | N |  | 8.64 | 67 |  |
| **74** | MDS | 60 | M | N |  |  |  |  |
| **75** | MDS | 80 | M | N |  |  |  |  |
| **76** | MDS | 77 | F | N |  |  |  |  |
| **77** | MDS | 69 | F | N |  |  |  |  |
| **78** | MDS | 67.2 | F | Y |  |  |  |  |
| **79** | MDS | 42 | F | Y | 3.3 | 8.5 | 20 | Hypo |
| **80** | AA | 51 | M | Y |  |  |  | Hypo |
| **81** | AA | 54 | F | Y | 2.6 | 10.1 | 33 | Hypo |
| **82** | AA | 48 | F | Y |  |  |  | Hypo |
| **83** | AA | 29 | M | Y | 1 | 6.6 | 240 | Hypo |

UPN: unidentified patient number; +6: trisomy 6; Hb: hemoglobin; WBC: White blood cells; pAML: primary acute myeloid leukemia; sAML: secondary acute myeloid leukemia; MDS: myelodysplastic neoplasm; AA: aplastic anemia; M: male; F: female; L: liter; g/dL: grams per deciliter; uL: microliter.

**Table S2. All cases of trisomy 6 from the literature included in the clinical analysis of the study.**

| **UPN** | **Diagnosis** | **Age (years)** | **Sex** | **Isolated +6** | **WBC (x10^9^/L)** | **Hb (g/dL)** | **Platelets (x10^3^/uL)** | **Bone marrow cellularity adjusted for age** |
| --- | --- | --- | --- | --- | --- | --- | --- | --- |
| **1** | sAML | 74 | M | Y |  |  |  |  |
| **2** | pAML | 50 | F | Y |  |  |  |  |
| **3** | pAML | 24 | F | Y |  |  |  |  |
| **4** | pAML | 81 | M | Y |  |  |  |  |
| **5** | pAML |  | F | Y |  |  |  |  |
| **6** | pAML | 55 | M | Y |  |  |  |  |
| **7** | pAML | 32 | F | Y |  |  |  |  |
| **8** | pAML | 37 | F | Y |  |  |  |  |
| **9** | pAML | 63 | F | Y | 4.8 | 13.5 | 6 | Hypo |
| **10** | pAML | 28 | F | Y | 4 | 11 | 53 | Normo |
| **11** | pAML | 66 | F | Y | 2.6 | 8.1 | 51 | Normo |
| **12** | pAML | 74 | M | Y | 29.4 | 9.3 | 26 | Hyper |
| **13** | pAML | 22 | M | Y | 4.4 | 12.5 | 238 | Normo |
| **14** | pAML | 40 | M | Y | 86.5 | 11.1 | 63 | Hyper |
| **15** | sAML | 37 | F | Y | 5.1 | 12.2 | 11 | Hypo |
| **16** | pAML | 41 | F | Y |  |  |  |  |
| **17** | pAML | NA | F | Y |  |  |  |  |
| **18** | pAML | 61 | F | Y |  |  |  |  |
| **19** | pAML | 51 | M | Y | 20.8 | 13.5 | 4 | Hyper |
| **20** | pAML | 25 | F | Y | 82.9 | 8 | 109 | Hyper |
| **21** | pAML | 82 | M | Y | 5.1 | 6.4 | 21 | Normo |
| **22** | pAML | 21 | F | Y | 56.3 | 5.4 | 85 | Hyper |
| **23** | pAML | 50 | F | Y |  | 8.5 | 11 | Hyper |
| **24** | pAML | 75 | M | Y | 103.8 | 7.3 | 80 |  |
| **25** | pAML | 43 | F | Y |  |  |  |  |
| **26** | pAML | 11 | M | Y |  |  |  |  |
| **27** | pAML | 8 | F | Y | 99 |  |  |  |
| **28** | pAML | 8 | M | Y |  |  |  |  |
| **29** | pAML | 2 | M | Y | 16.4 | 7.6 | 6 |  |
| **30** | pAML | 8 | F | Y |  |  |  |  |
| **31** | pAML | 14 | M | Y | 244 | 5.7 | 52 | Hyper |
| **32** | pAML | 1 | F | Y | 30.5 |  |  |  |
| **33** | MDS | 37 | F | Y | 2.1 | 9.9 | 56 | Hypo |
| **34** | MDS | 22 | M | Y | 2.1 | 9.9 | 56 | Hypo |
| **35** | MDS | 43 | M | Y | 3.2 | 8.3 | 33 | Hypo |
| **36** | MDS | 74 | M | Y |  |  |  |  |
| **37** | MDS | 43 | F | Y |  |  |  |  |
| **38** | MDS | 2 | M | Y | 16.4 | 7.6 | 6 |  |
| **39** | MDS | 41 | F | Y |  |  |  |  |
| **40** | MDS | 49 | F | Y |  |  |  |  |
| **41** | MDS | 34 | M | Y | 3.3 | 7.5 | 16 | Hypo |
| **42** | MDS | 48 | M | Y |  |  |  |  |
| **43** | MDS | 51 | F | Y |  |  |  |  |
| **44** | MDS | 34 | M | Y |  | 8 | 10 | Hypo |
| **45** | AA | 34 | M | Y |  | 7.5 | 16 | Hypo |
| **46** | AA | 42 | M | Y | 1.8 | 7.5 | 11 | Hypo |
| **47** | AA | 31 | F | Y | 2.9 | 10.1 | 13 | Hypo |
| **48** | AA | 33 | M | Y | 6.6 | 12.1 | 25 | Hypo |
| **49** | AA | 24 | M | Y |  |  |  | Hypo |
| **50** | AA | 51 | F | Y |  |  |  | Hypo |
| **51** | AA | 36 | F | Y |  |  |  | Hypo |
| **52** | AA | 50 | F | Y | 2 | 6.7 | 13 | Hypo |
| **53** | AA | 54 | F | Y | 2.1 | 5.3 | 14 | Hypo |
| **54** | AA | 40 | F | Y |  |  |  | Hypo |

UPN: unidentified patient number; +6: trisomy 6; Hb: hemoglobin; WBC: White blood cells; pAML: primary acute myeloid leukemia; sAML: secondary acute myeloid leukemia; MDS: myelodysplastic neoplasm; AA: aplastic anemia; M: male; F: female; L: liter; g/dL: grams per deciliter; uL: microliter.

| Variables | Original Iso +6 AML | Literature Iso +6 AML | P-value |
| --- | --- | --- | --- |
|  | N=16 | N=32 |  |
| WBC, median (IQR) | 20.1 (2.3-61.3) | 25.1 (4.9-85.6) | 0.3506 |
| Hemoglobin, median (IQR) | 7.8 (7.0-8.03) | 8.5 (7.3-12.2) | 0.1148 |
| Platelets, median (IQR) | 60.0 (36.8-92.0) | 51.0 (11.0-80.0) | 0.4545 |
| Bone marrow blasts, median (IQR) | 55.0 (37.3-63.0) | 50.1 (23.6-77.8) | 0.9322 |
| Underwent allo HSCT, n (%) |  |  |  |
| Yes | 4 (66.7%) |  |  |
| No | 2 (33.3%) |  |  |
| Relapse after HSCT, n (%) | 0 (0.0%) |  |  |
| Survival in months | 32.3 | 22 | 0.6110 |

**Table S3. Comparison of the characteristics of isolated trisomy 6 AML patients obtained from our original cohort versus the ones reported in the literature.**

Iso +6: Isolated trisomy 6; AML: Acute myeloid Leukemia; IQR: interquartile range; WBC: White blood cells; HSCT: hematopoietic stem cell transplant.

**Table S4. Set of genes sequenced in our targeted panels.**

| *ASXL1* | *BCOR* | *BCORL1* | *CALR* | *CBL* | *CEBPA* |
| --- | --- | --- | --- | --- | --- |
| *CSF1R* | ***CUX1*** | ***DNMT3A*** | ***EED*** | ***ETV6*** | ***EZH2*** |
| *FLT3* | ***GATA2*** | ***IDH1*** | ***IDH2*** | ***JAK2*** | ***KDM6A*** |
| *KIT* | ***KMT2A*** | ***KRAS*** | ***LUC7L2*** | ***MECOM*** | ***NF1*** |
| *NPM1* | ***NRAS*** | ***PHF6*** | ***PRPF8*** | ***PTPN11*** | ***RAD21*** |
| *RUNX1* | ***SETBP1*** | ***SF3B1*** | ***SMC1*** | ***SMC3*** | ***SRSF2*** |
| *STAG2* | ***STAT3*** | ***SUZ12*** | ***TET2*** | ***TP53*** | ***U2AF1*** |
| *WT1* | ***ZRSR2*** |  |  |  |  |

**Table S5. Frequency of specific gene mutations in iso +6 and non-iso +6 AML.**

| Mutations | Iso +6 AML (%) | Non-Iso +6 AML (%) |
| --- | --- | --- |
| N | **16** | **46** |
| *ASXL1* | 6.3 | 13.0 |
| *BCOR* | 6.3 | 4.4 |
| *BCORL1* | 6.3 | 2.2 |
| *CEBPA* | 6.3 | 0 |
| *CUX1* | 6.3 | 4.4 |
| *DNMT3A* | 18.8 | 4.4 |
| *ETV6* | 0 | 0 |
| *EZH2* | 6.3 | 0 |
| *FLT3* | 25 | 0 |
| *GATA2* | 0 | 0 |
| *IDH1* | 6.3 | 6.5 |
| *IDH2* | 0 | 4.4 |
| *JAK2* | 0 | 0 |
| *KIT* | 0 | 0 |
| *KRAS* | 6.3 | 6.5 |
| *NF1* | 0 | 2.2 |
| *NPM1* | 0 | 0 |
| *NRAS* | 6.3 | 8.7 |
| *PPM1D* | 0 | 0 |
| *PTPN11* | 6.3 | 2.2 |
| *RAD21* | 6.3 | 4.4 |
| *RUNX1* | 0 | 8.7 |
| *SETBP1* | 0 | 0 |
| *SF3B1* | 0 | 0 |
| *SRSF2* | 0 | 8.7 |
| *STAG2* | 0 | 0 |
| *TET2* | 18.8 | 8.7 |
| *TP53* | 0 | 41.3 |
| *U2AF1* | 0 | 2.2 |
| *WT1* | 0 | 0 |
| *ZRSR2* | 6.3 | 0 |

Iso +6: isolated trisomy 6; AML: acute myeloid leukemia.

**Table S6. HLA sequencing for mutations and allelic loss in +6 MDS and AML patients with available bone marrow samples.**

| **UPN** | **Diagnosis** | **HLA mutations** | **HLA allelic losses** | **Lost HLA allele** |
| --- | --- | --- | --- | --- |
| **#1** | iso +6 MDS | None | None | None |
| **#2** | iso +6 MDS | None | None | None |
| **#3** | iso +6 MDS | None | None | None |
| **#4** | iso +6 AML | None | Present | HLA-DRB1*07:01:01:09 |
| **#5** | iso +6 AML | None | Present | HLA-DRB1*04:08:01:03 |
| **#6** | iso +6 AML | None | None | None |
| **#7** | iso +6 AML | None | None | None |
| **#8** | iso +6 AML | None | None | None |

UPN: unidentified patient number; HLA: Human leukocyte antigen; Iso +6: Isolated trisomy 6; MDS: myelodysplastic neoplasm; AML: acute myeloid leukemia

**Figure S1. Kaplan-Meier survival analysis comparing overall survival in isolated trisomy AML, non-isolated trisomy 6 AML, and normal karyotype AML.**

**
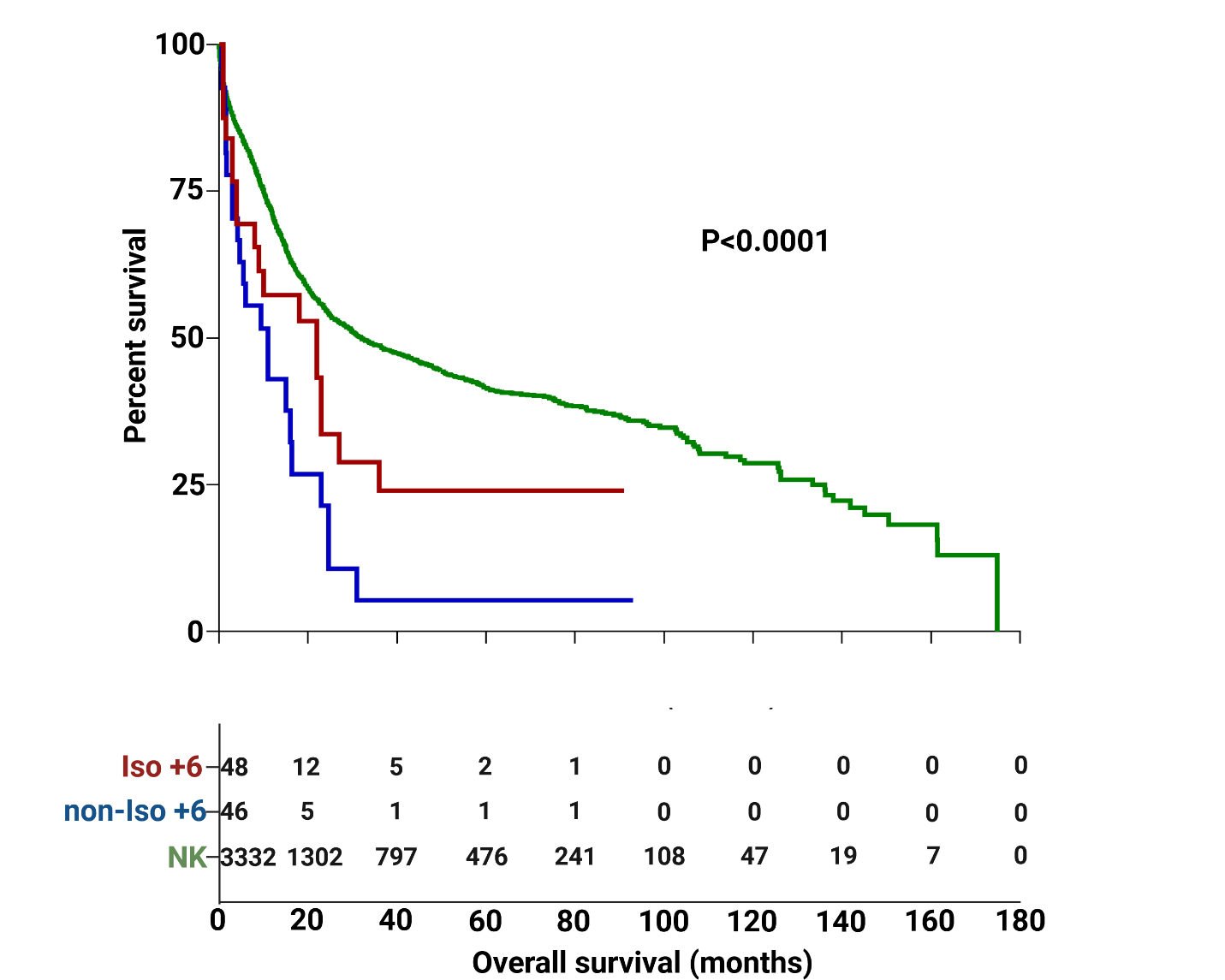
**

**References**

1. Tyner JW, Tognon CE, Bottomly D, Wilmot B, Kurtz SE, Savage SL*, et al.* Functional genomic landscape of acute myeloid leukaemia. *Nature* 2018 Oct; **562**(7728)**:** 526-531.

2. Ley TJ, Miller C, Ding L, Raphael BJ, Mungall AJ, Robertson A*, et al.* Genomic and epigenomic landscapes of adult de novo acute myeloid leukemia. *N Engl J Med* 2013 May 30; **368**(22)**:** 2059-2074.

3. Papaemmanuil E, Gerstung M, Bullinger L, Gaidzik VI, Paschka P, Roberts ND*, et al.* Genomic Classification and Prognosis in Acute Myeloid Leukemia. *N Engl J Med* 2016 Jun 9; **374**(23)**:** 2209-2221.

4. Bersanelli M, Travaglino E, Meggendorfer M, Matteuzzi T, Sala C, Mosca E*, et al.* Classification and Personalized Prognostic Assessment on the Basis of Clinical and Genomic Features in Myelodysplastic Syndromes. *J Clin Oncol* 2021 Apr 10; **39**(11)**:** 1223-1233.

5. Gurnari C, Pagliuca S, Prata PH, Galimard JE, Catto LFB, Larcher L*, et al.* Clinical and Molecular Determinants of Clonal Evolution in Aplastic Anemia and Paroxysmal Nocturnal Hemoglobinuria. *J Clin Oncol* 2023 Jan 1; **41**(1)**:** 132-142.

6. Mitelman F, Johansson B, Mertens F. Mitelman Database of Chromosome Aberrations and Gene Fusions in Cancer [cited; Available from: <https://mitelmandatabase.isb-cgc.org>

7. Shaffer LG, Tommerup N. *ISCN 2005 : an international system for human cytogenetic nomenclature (2005) : recommendations of the International Standing Committee on Human Cytogenetic Nomenclature*. Karger: Basel ;, 2005.

8. Awada H, Durmaz A, Gurnari C, Kishtagari A, Meggendorfer M, Kerr CM*, et al.* Machine learning integrates genomic signatures for subclassification beyond primary and secondary acute myeloid leukemia. *Blood* 2021 Nov 11; **138**(19)**:** 1885-1895.

9. Gurnari C, Pagliuca S, Guan Y, Adema V, Hershberger CE, Ni Y*, et al.* TET2 mutations as a part of DNA dioxygenase deficiency in myelodysplastic syndromes. *Blood Adv* 2022 Jan 11; **6**(1)**:** 100-107.

10. Wang K, Li M, Hakonarson H. ANNOVAR: functional annotation of genetic variants from high-throughput sequencing data. *Nucleic Acids Res* 2010 Sep; **38**(16)**:** e164.

11. Hirsch CM, Przychodzen BP, Radivoyevitch T, Patel B, Thota S, Clemente MJ*, et al.* Molecular features of early onset adult myelodysplastic syndrome. *Haematologica* 2017 Jun; **102**(6)**:** 1028-1034.

12. Patro R, Duggal G, Love MI, Irizarry RA, Kingsford C. Salmon provides fast and bias-aware quantification of transcript expression. *Nat Methods* 2017 Apr; **14**(4)**:** 417-419.

13. Soneson C, Love MI, Robinson MD. Differential analyses for RNA-seq: transcript-level estimates improve gene-level inferences. *F1000Res* 2015; **4:** 1521.
